# Selenium nanoparticles and glutathione synergistically enhance salt tolerance in soybean via the jasmonic acid pathway and arbutin-regulated rhizosphere microbiota

**DOI:** 10.1016/j.abiote.2026.100022

**Published:** 2026-01-15

**Authors:** Yanxi Chen, Shuai Zhao, Xuanxuan Ma, Li Ling, Pengxin Du, Xu Liu, Yuhao Yao, Qibin Ma, Yanbo Cheng, Yingxiang Wang, Jian Wei, Hai Nian, Tengxiang Lian

**Affiliations:** a Guangdong Basic Research Center of Excellence for Precise Breeding of Future Crops, Guangdong Laboratory for Lingnan Modern Agriculture, Guangdong Provincial Key Laboratory for the Development Biology and Environmental Adaptation of Agricultural Organisms, South China Institute for Soybean Innovation Research, College of Agriculture, South China Agricultural University, Guangzhou, 510642, China; b State Key Laboratory of Desert and Oasis Ecology, Xinjiang Institute of Ecology and Geography, Chinese Academy of Sciences, Urumqi, 830011, China; c Northern Key Laboratory of Saline-tolerant Soybean Breeding, Ministry of Agriculture and Rural Areas, Jilin Agricultural University, Changchun, 130118, China

**Keywords:** SeNPs@CS, GSH, Soybean, Salt stress, Jasmonic acid, Arbutin, Rhizosphere microbiome

## Abstract

Soil salinity threatens agriculture worldwide. Nano-fertilizers offer a promising strategy to enhance tolerance to salinity and other stresses in crops, but their field performance is sometimes unpredictable, potentially due to complex interactions within the plant–microbe holobiont. Here, we designed chitosan-stabilized selenium nanoparticles (SeNPs@CS) as a novel nano-fertilizer. SeNPs@CS exhibited a uniform size (∼109.8 nm) and a positively charged surface (+14.7 mV), which confers good adhesion to plant tissues. Due to their good biocompatibility and small size, SeNPs@CS can be readily absorbed and utilized by plant leaves. When SeNPs@CS were combined with glutathione (GSH) to form a nanocomposite (SeG), they significantly promoted plant growth and enhanced salt tolerance in soybean (*Glycine max*). Multi-omics analyses revealed that SeG activates jasmonic acid (JA) pathways in the plant and remodels the root metabolic profile, leading to the enrichment of arbutin, a key signaling molecule, in the rhizosphere. This metabolic shift recruits and enriches beneficial salt-tolerant microbes, including Bacillus and Streptomyces, thereby establishing a protective microbiome. Treatment of plants with a synthetic microbial community (SynCom) composed of these elite strains, in combination with arbutin, reproduced the salt tolerance phenotype conferred by SeG treatment. Therefore, SeG improves salt tolerance in soybean via activation of the JA defense pathway and arbutin-driven recruitment of salt-tolerant rhizosphere microorganisms. Together, these two mechanisms enhance plant resilience under salt stress. This multi-kingdom synergistic mechanism for alleviating stress provides a new paradigm for developing smart agricultural inputs that target the plant holobiont to improve crop resilience.

## 1 Introduction

The leguminous crop soybean (*Glycine max*) supplies protein and oils for human nutrition and industrial applications, playing a vital role in global food security and industrial output [1,2]. However, soil salinization poses a growing threat to soybean yield and quality [3]. Salt stress induces osmotic and ionic imbalance in plants and simultaneously promotes the excessive accumulation of reactive oxygen species (ROS), leading to oxidative damage [4,5]. These stresses disrupt key physiological processes, including photosynthesis and stomatal regulation, thereby impairing the plant's ability to maintain osmotic homeostasis and exacerbating water loss and ion toxicity [6,7]. In parallel, excess salt disrupts the interactions between plants and beneficial soil microorganisms, further weakening the ability of the plant to withstand environmental stress and exacerbating the negative effects of salt on soybean growth [8]. Traditional methods for managing soil salinization, such as irrigation and the use of chemical amendments, are costly and environmentally harmful [9]. Over time, these approaches are unsustainable, contributing to ecological degradation and hindering progress toward sustainable agricultural systems. Therefore, there is an urgent need for innovative, environmentally friendly strategies that enhance the salt stress tolerance of soybeans, helping secure agricultural productivity and environmental health.

Redox homeostasis is a crucial aspect of stress tolerance and enhancing plant mechanisms for scavenging ROS may enhance tolerance to soil salinity. For example, in the ascorbic acid (AsA)-glutathione (GSH) cycle, GSH reacts with ROS to form glutathione disulfide, which is regenerated to the active GSH form by reacting with AsA. GSH treatment reduces ROS accumulation in rice (*Oryza sativa*) root tips under aluminum stress, maintaining redox balance; it also promotes the biosynthesis of GSH-derived phytochelatins, which transport aluminum ions to the vacuole, thereby reducing the cytosolic aluminum ion concentration and ensuring normal root growth [10].

Importantly, the efficiency of plant antioxidant defenses under stress conditions is not determined by individual redox components alone, but rather by the coordinated functioning of redox-active molecules and redox-related enzymes. In this context, glutathione serves not only as a direct ROS scavenger, but also as a central redox buffer and an essential substrate for multiple antioxidant enzymes. The capacity of the GSH-dependent antioxidant system is therefore closely linked to the availability and functionality of its associated catalytic components, which often require specific mineral cofactors for their enzymatic activity, particularly those involved in peroxide detoxification.

For example, selenium is a critical component of the active site of glutathione peroxidase (GSH-Px) [11], an enzyme that catalyzes the reduction of hydrogen peroxide using GSH as an electron donor [12]. Selenium nanoparticles (SeNPs), as a zero-valent nanomaterial, can be converted within plant tissues into bioactive selenium forms that participate in these antioxidant enzyme systems. In leaves of salt-stressed rapeseed (*Brassica napus* subsp. *napus*), SeNPs treatment decreased ROS accumulation, thereby maintaining redox homeostasis, reduced proline accumulation by regulating osmotic balance, and promoted the conversion of starch into soluble sugars, providing additional energy for plant growth under salt stress [13]. However, the application of bare SeNPs is constrained by their propensity for aggregation, resulting in reduced biological activity. Chitosan-stabilized SeNPs (SeNPs@CS) leverage the excellent biocompatibility and electrostatic stabilizing capacity of chitosan to enhance both dispersibility and selenium delivery efficiency. This modification confers improved environmental stability and broader application potential compared with conventional SeNPs.

The activation of the antioxidant system in plants under stress conditions is often accompanied by other metabolic changes [14,15]. In addition to enhancing stress resistance, stress-induced metabolic changes help shape the rhizosphere microbial community by altering the metabolites secreted by the root. Some of these metabolites act as signaling molecules that attract beneficial microbes to the rhizosphere [[16], [17], [18]]. Rhizosphere microbes, often referred to as the plant's “second genome” [19], bolster plant resilience to environmental stresses [[20], [21], [22]]. For instance, under salt stress, soybean secretes xanthine into the rhizosphere. This restructures the rhizosphere microbial community, enriching beneficial *Pseudomonas* strains by activating *Pseudomonas* genes involved in chemotaxis and flagellar assembly [23]. Similarly, plants in the Cucurbitaceae enhance salt tolerance by recruiting specific root-associated bacterial communities that mitigate the stress, with effects that are greater than the effects of the individual strains [24].

Treatment with various substances may amplify this rhizosphere defense mechanism. For example, in pepper (*Capsicum annuum*), exogenously applied SeNPs induces significant shifts in the rhizosphere microbiome composition and host metabolite profiles through multiple signaling pathways and recruits beneficial microbial taxa such as Gammaproteobacteria and Alphaproteobacteria to alleviate cadmium stress [25]. Likewise, SeNPs promote the enrichment of beneficial *Bacillus* species in the maize (*Zea mays*) rhizosphere while triggering metabolic reprogramming in the host via multiple cross-kingdom signaling pathways [26]. Together, these findings point to a complex regulatory network involving SeNPs, host metabolic responses, and rhizosphere microbial interactions, offering a new perspective on plant survival strategies under abiotic stress.

SeNPs and GSH act synergistically to maintain plant redox homeostasis [27], but the exact mechanisms remain unclear. Building on these findings, we reasoned that the combined application of SeNPs and GSH would activate the antioxidant-metabolic network and associated metabolic changes in soybean, thereby enhancing plant–microbe defenses and improving salt stress tolerance. In this study, we synthesized SeNPs@CS via chemical reduction and employed a multi-omics approach integrating transcriptomics, metabolomics, and microbial community analysis to comprehensively investigate the responses of soybean to salt stress following combined treatment with SeNPs@CS and GSH. Specifically, we examined changes in gene expression, metabolic profiles, and the composition and functional traits of rhizosphere microbial communities. In parallel, we isolated and cultured enriched microbial taxa to test their roles in facilitating soybean acclimation to saline conditions. Together, our findings bridge a critical knowledge gap regarding the effects of SeNPs and GSH in enhancing salt tolerance in soybean, with practical implications for improving crop resilience in saline environments.

## 2 Results

### 2.1 Morphological characterization of SeNPs@CS

**Fig. 1:**
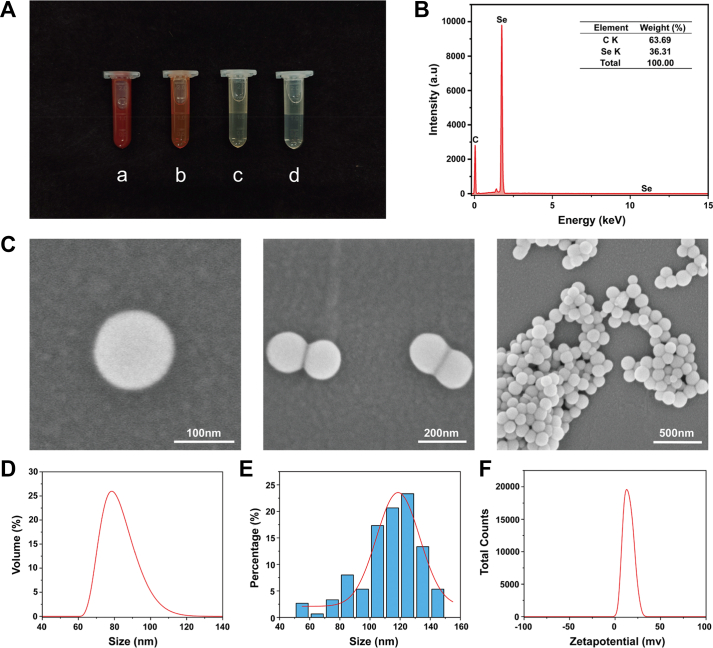
Physicochemical characterization of SeNPs@CS. **A** Photographs of SeNPs@CS solutions at different concentrations (a: 1500, b: 150, c: 10, and d: 5 mg/L). **B** Energy-dispersive X-ray spectroscopy (EDS) spectrum showing the elemental composition of SeNPs@CS. **C** Scanning electron microscopy (SEM) images of SeNPs@CS at different magnifications. **D** Hydrodynamic size distribution measured by dynamic light scattering (DLS). **E** Particle size distribution determined from SEM images. **F** Zeta potential profile of SeNPs@CS in aqueous solution.

Using chemical reduction of selenious acid (H_2_SeO_3_), we produced a stable, dark-red suspension of SeNPs@CS [28]; the uniform hue of this suspension implies that the H_2_SeO_3_ was completely converted to elemental Se (Fig. 1A). Energy-dispersive X-ray spectroscopy confirmed the homogeneous distribution of Se, with a single intense peak (Fig. 1B). Scanning electron microscopy (SEM) revealed discrete, spherical particles with limited aggregation (Fig. 1C) and a mean diameter of 109.8 nm, which closely matches the hydrodynamic diameter (101.5 nm) measured by dynamic light scattering (Fig. 1D and E). Zeta potential measurements showed a positive surface charge of +14.7 mV (Fig. 1F). These results demonstrate that the chemical-reduction method successfully generated monodisperse, positively charged, colloidally stable SeNPs@CS suitable for subsequent analysis.

### 2.2 Optimization of SeNPs@CS and GSH treatment concentrations and biosafety evaluation

To evaluate the biosafety of SeNPs@CS and determine the appropriate treatment concentrations, we examined the effects of different SeNPs@CS concentrations on soybean seed germination and early seedling growth (Fig. S1A and B). No significant reduction in germination rate was detected in any SeNPs@CS treatment group, with all groups maintaining germination percentages above 76.7 %. Even at the highest concentration (40 mg/L), SeNPs@CS did not induce any visible phytotoxicity, indicating that the material is safe for soybean plants during early development.

Subsequently, we performed a foliar-dose screen with five SeNPs@CS levels (0, 5, 10, 20, and 40 mg/L) and six GSH levels (0, 10, 25, 50, 100, and 200 mg/L) to identify the optimal concentrations. Treatment with 10 mg/L SeNPs@CS produced the greatest increase in growth, increasing aboveground and belowground biomass by 33.2 % and 49.5 %, respectively, compared to the control (Fig. S1C and D). Notably, high concentrations of SeNPs@CS had no significant inhibitory effects on seedling growth, indicating that the selected concentration range of SeNPs is safe for soybean growth. In addition, 25 mg/L GSH yielded the highest aboveground and belowground biomass (Fig. S1E and F). These results demonstrate that SeNPs@CS is biosafe across the tested concentration range and that both SeNPs@CS and GSH exhibit clear optimal doses for promoting soybean seedling growth. We selected 10 mg/L SeNPs@CS and 25 mg/L GSH for subsequent combined treatments.

### 2.3 SeNPs@CS and GSH synergistically promote soybean growth

**Fig. 2:**
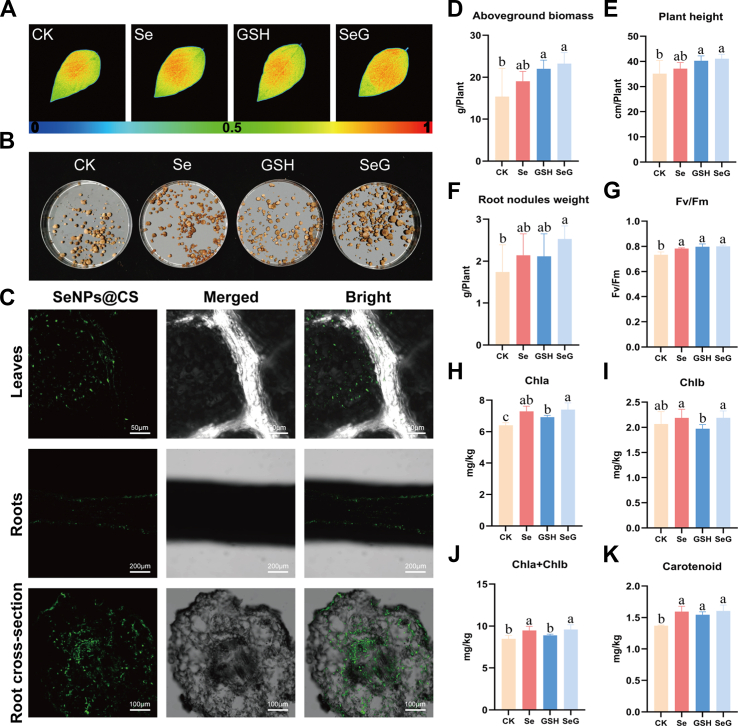
Effects of SeNPs, glutathione, and their combination on soybean physiological parameters and confocal fluorescence imaging of FITC-labeled SeNPs@CS. **A** Chlorophyll fluorescence imaging was used to visualize Fv/Fm patterns in typical leaves, with the scale bar indicating Fv/Fm values from 0 (blue) to 1 (red). **B** Images of root nodules in soybean plants under different treatments. **C** Confocal images of soybean leaves, roots, and root cross-sections after treatment with FITC-labeled SeNPs@CS. Green fluorescence represents FITC-labeled SeNPs@CS. **D-F** Quantitative analysis of aboveground biomass (D), plant height (E), and root nodule weight (F), *n* = 6. **G** Maximum quantum efficiency of PSII (Fv/Fm), *n* = 3. **H–K** Contents of photosynthetic pigments including chlorophyll *a* (H), chlorophyll *b* (I), total chlorophyll (J), and carotenoids (K), *n* = 9. Different letters above bars indicate statistical differences among treatments (*P* < 0.05, LSD test).

To evaluate the physiological effects of SeNPs@CS (10 mg/L), GSH (25 mg/L), and their combination (SeG) on soybean plants under non-stress conditions, we treated soybean seedlings with these compounds via foliar application. Compared to Se, GSH, and control (CK) treatments, SeG-treated soybean seedlings displayed markedly enhanced growth performance (Fig. S2A–D, Fig. 2A). Given the enhanced root nodule development observed after foliar SeG application (Fig. 2B), we hypothesized that SeNPs@CS could be translocated from leaves to the root zone to exert their effects. Confocal imaging with FITC-labeled SeNPs@CS confirmed rapid absorption through leaves and systemic transport to roots (Fig. 2C), likely due to their nanoscale size and positive surface charge, which facilitate cellular penetration and long-distance movement [[29], [30], [31]].

SeG-treated plants exhibited superior performance: aboveground biomass, plant height, and root nodule weight increased by 21 %, 18 %, and 46 %, respectively (Fig. 2D–F), and the maximum quantum efficiency (Fv/Fm) of PSII improved by 9.1 %, surpassing the treatments with Se or GSH alone (Fig. 2G). SeG also increased chlorophyll *a*, chlorophyll *b*, total chlorophyll, and carotenoid contents (Fig. 2H–K). These results demonstrate that foliar-applied SeNPs@CS can translocate to the root zone and that its combination with GSH enhances both shoot and root growth in soybean under non-stress conditions.

### 2.4 SeNPs@CS and GSH cooperatively strengthen the antioxidant network

Se, GSH, and SeG treatments modulated redox metabolism, with SeG inducing the most comprehensive changes (Fig. S3). SeG enhanced antioxidant enzyme activities, increasing superoxide dismutase (SOD), peroxidase (POD), and catalase (CAT) activities by 19.4 %, 15.7 %, and 12.7 %, respectively, while decreasing malondialdehyde (MDA) content by 54 % relative to the control (Fig. S3A–D). Total antioxidant capacity increased by 9.5 % compared to the control (Fig. S3E). The activity of phenylalanine ammonia-lyase (PAL), a key phenylpropanoid enzyme [32,33], increased under all treatments (Fig. S3F). Among members of the AsA-GSH cycle, SeG enhanced ascorbate peroxidase (APX) and monodehydroascorbate reductase (MDHAR) activities by 35.7 % and 9.7 %, respectively (Fig. S3G and H). Glutathione peroxidase (GSH-Px) activity increased by 27.2 %, 25.4 %, and 24.2 % in response to Se, GSH, and SeG treatments, respectively (Fig. S3I), with SeG-treated plants containing 16.7 % more reduced GSH than the control (Fig. S3J). These results indicate that Se, GSH, and especially SeG enhance both enzymatic and non-enzymatic antioxidant pathways, leading to a strengthened redox-protective capacity in soybean under non-stress conditions.

### 2.5 Effects of individual and combined SeNPs@CS and GSH treatments on soybean growth under salt stress

**Fig. 3:**
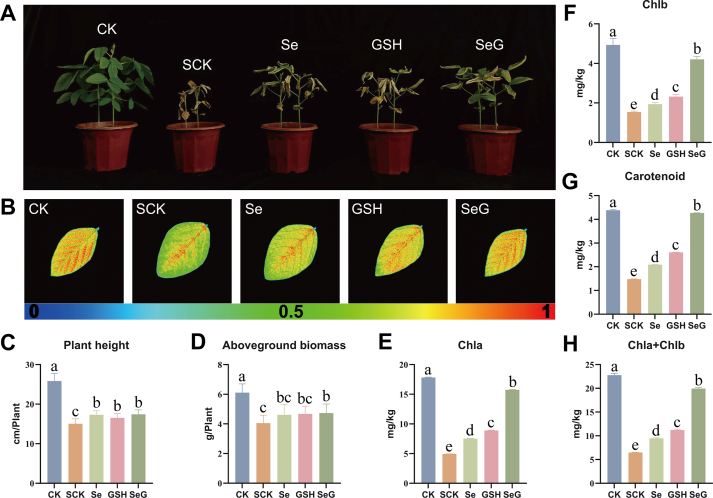
Effects of Se, GSH, and SeG on soybean morphology and photosynthetic traits under salt stress. **A** Phenotypic differences among CK, SCK, Se, GSH, and SeG treatments after salt exposure. **B** Fv/Fm imaging of representative leaves visualized using chlorophyll fluorescence imaging; the scale bar represents Fv/Fm values ranging from 0 (blue) to 1 (red). **C**, **D** Plant height and aboveground biomass, *n* = 12. **E**, **F** Chlorophyll *a* (Chla) and chlorophyll *b* (Chlb) contents, *n* = 6. **G** Carotenoid content. **H** Total chlorophyll content (Chla + Chlb), *n* = 6. Different letters above bars indicate statistical differences among treatments (*P* < 0.05, LSD test).

Salt stress severely suppressed plant growth, with salt-treated plants (SCK) showing stunting and wilting (Fig. 3A and Fig. S4A). Foliar application of Se, GSH, and SeG alleviated these symptoms, with SeG being the most effective. Chlorophyll fluorescence imaging showed that SeG enhanced Fv/Fm (Fig. 3B). Compared with CK, SCK plants exhibited pronounced growth inhibition, with plant height, aboveground biomass, root length, and belowground biomass reduced by 41.9 %, 33.7 %, 34.9 %, and 32.1 %, respectively. Foliar application of Se, GSH, and SeG alleviated these negative effects, with SeG exhibiting the strongest effects. Specifically, SeG treatment increased plant height, aboveground biomass, root length, and belowground biomass by 16.0 %, 16.9 %, 40.9 %, and 28.8 %, respectively, compared to SCK, bringing the growth status of plants closer to that of the control (Fig. 3C and D; Fig. S4B and C). Pigment levels mirrored these trends (Fig. 3E–H). Total chlorophyll content dropped to 28.5 % of control levels under salt stress. Se and GSH increased the total chlorophyll content to 41.6 % and 49.3 % of control levels, respectively, whereas SeG restored this value to 87.6 %, approaching the unstressed baseline. The levels of chlorophyll *a*, chlorophyll *b*, and carotenoids followed a similar pattern, supporting the strong pigment-stabilizing effect of SeG [27]. These findings indicate that although Se and GSH partially alleviated salt-induced damage, SeG had the most pronounced restorative effects on growth traits and photosynthetic pigment accumulation.

### 2.6 SeNPs@CS and GSH strengthen antioxidant defense under salt stress

**Fig. 4:**
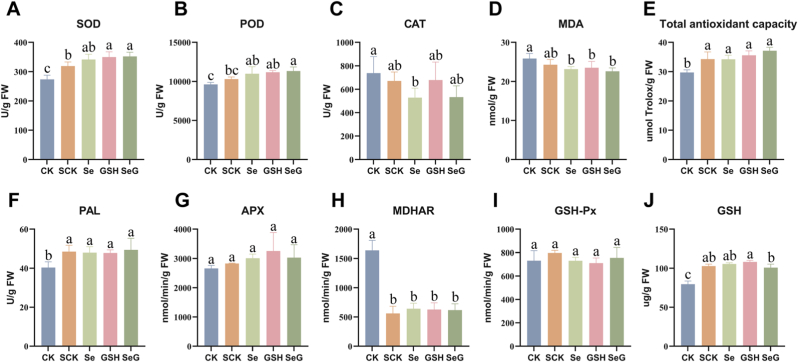
Antioxidant enzyme activities and related indicators in soybean leaves under salt stress. **A**-**J** Levels of SOD (A), POD (B), CAT (C), MDA (D), Total antioxidant capacity (E), PAL (F), APX (G), MDHAR (H), GSH-Px (I), and GSH (J) after CK, SCK, Se, GSH, and SeG treatments, *n* = 3. Different letters above bars indicate statistical differences among treatments (*P* < 0.05, LSD test).

Salt treatment disturbed the redox balance of soybean plants (Fig. 4A–J). SOD and POD activities were 16.7 % and 7.0 % higher in SCK, respectively, than in CK (Fig. 4A and B). Se, GSH, and SeG treatments further enhanced the activities of these enzymes. SeG increased SOD and POD activities by 10.3 % and 10.0 %, respectively, compared to SCK. CAT activity declined by 26 % under Se or SeG treatment but increased slightly in plants treated with GSH (Fig. 4C). Compared with CK, SCK showed no significant change in MDA content, whereas MDA levels significantly decreased under Se, GSH, and SeG treatments, decreasing by 10.5 %, 9.2 %, and 12.6 %, respectively (Fig. 4D). Total antioxidant capacity increased by 15.3 % in SCK compared with CK; moreover, Se, GSH, and SeG treatments led to additional increases of 3.3 %, 7.1 %, and 8.1 % relative to SCK, respectively (Fig. 4E). PAL activity increased by 19.9 % in SCK and 23.8 % in SeG-treated plants (Fig. 4F). MDHAR activity was markedly suppressed under salt stress and showed only slight recovery across treatments (Fig. 4H). In addition, GSH content was lowest in the SeG group (Fig. 4J). These results indicate that salt stress substantially altered antioxidant enzyme activities and redox indicators. SeG produced the strongest overall improvements among treatments, particularly in enhancing SOD activity, POD activity, and total antioxidant capacity.

### 2.7 Multi-omics insights into SeG-mediated alleviation of salt stress in soybean

**Fig. 5:**
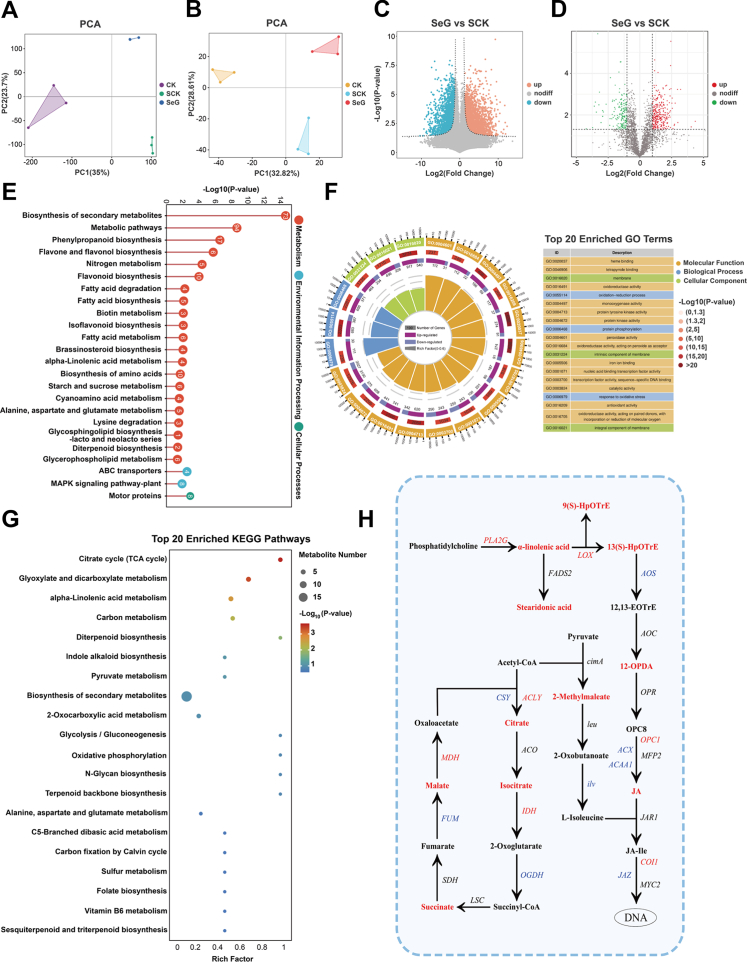
Multi-omics analysis of molecular responses to SeG treatment under salt stress. **A**, **B** Principal component analysis (PCA) of transcriptomic (A) and metabolomic (B) profiles across CK, SCK, and SeG groups. **C** Volcano plot of differentially expressed genes (DEGs) in the SeG vs SCK comparison (|Log_2_FC| > 2, *P* < 0.05). **D** Volcano plot of differentially expressed metabolites (DEMs) in the SeG vs SCK comparison (|Log_2_FC| > 1, *P* < 0.05). **E** KEGG pathway enrichment of DEGs in the SeG vs SCK group shown as a lollipop plot. Red, blue, and green indicate metabolism, environmental information processing, and cellular processes. **F** Circular visualization of the top 20 significantly enriched GO terms in the SeG vs SCK group based on transcriptomic analysis. **G** KEGG enrichment analysis of DEMs in the SeG vs SCK group, shown as a bubble plot. Dot size represents the number of enriched metabolites, and color indicates statistical significance. **H** Reconstruction of the α-linolenic acid metabolism and JA biosynthesis pathway based on integrated omics data. Red and blue indicate significantly upregulated and downregulated genes/metabolites, respectively. Black labels represent those not significantly altered or undetected.

To elucidate the molecular mechanisms underlying the alleviation of salt stress by SeG, we performed integrated transcriptomic and metabolomic analyses on soybean root samples from three treatment groups (CK, SCK, and SeG), with three biological replicates per group. Principal component analysis (PCA) of the transcriptomic data revealed that PC1 and PC2 accounted for 35.0 % and 23.7 % of the total variance, respectively, indicating substantial transcriptional divergence among treatments (Fig. 5A). Similarly, PCA of metabolomic profiles showed that PC1 and PC2 explained 32.8 % and 28.6 % of the variance, respectively, demonstrating consistent separation and significant metabolic changes across treatments (Fig. 5B).

Differential gene expression analysis (|Log_2_(fold change)| ≥ 2, *P* < 0.05) identified 4987 differentially expressed genes (DEGs) in the SCK vs. CK comparison (1522 upregulated and 3465 downregulated), 3189 DEGs in SeG vs. CK (998 upregulated and 2189 downregulated), and 4510 DEGs in SeG vs. SCK (2710 upregulated and 1800 downregulated)(Fig. 5C; Fig. S5A and B). Based on the thresholds of |Log_2_(fold change)| ≥ 1 and *P* < 0.05, a total of 540, 613, and 454 differentially accumulated metabolites (DAMs) were detected in the SCK vs. CK, SeG vs. CK, and SeG vs. SCK comparisons, respectively. Among them, 254, 365, and 291 metabolites were upregulated, while 286, 248, and 163 metabolites were downregulated (Fig. 5D; Fig. S5C and D).

We conducted KEGG enrichment analysis of the upregulated DEGs to characterize the transcriptional responses triggered by SeG under salt stress (Fig. 5E; Fig. S6A and B). In the SeG vs. SCK comparison, the top 10 enriched pathways were biosynthesis of secondary metabolites, metabolic pathways, phenylpropanoid biosynthesis, flavone and flavonol biosynthesis, nitrogen metabolism, flavonoid biosynthesis, motor proteins, ABC transporters, fatty acid degradation, and fatty acid biosynthesis. GO enrichment analysis identified the functional categories associated with these transcriptional changes: we generated a circle plot highlighting the top 20 significantly enriched GO terms in SeG vs. SCK(Fig. 5F–Table S1). Among these, antioxidant-related terms included oxidoreductase activity (GO:0016491); oxidation-reduction process (GO:0055114); peroxidase activity (GO:0004601); oxidoreductase activity; acting on peroxide as acceptor (GO:0016684); response to oxidative stress (GO:0006979); antioxidant activity (GO:0016209); and oxidoreductase activity, acting on paired donors, with incorporation or reduction of molecular oxygen (GO:0016705). Notably, genes within these antioxidant-related pathways showed a pronounced bias toward upregulation rather than downregulation, reflecting an overall activation of antioxidant responses. The results indicate that SeG treatment activates the antioxidant pathways in soybean under salt stress.

To complement the findings from transcriptome analysis, we performed KEGG enrichment analysis of the upregulated DAMs to identify metabolically active pathways induced by SeG. Across all three comparison groups, DAMs were commonly enriched in basic metabolic categories such as global and overview maps, amino acid metabolism, and biosynthesis of secondary metabolites (Fig. S7A–C), indicating that salt stress and SeG treatment both induced widespread metabolic reprogramming in soybean roots.

Bubble plot analysis revealed that enriched pathways varied between comparisons, reflecting treatment-specific metabolic signatures (Fig. 5G and Fig. S7D and E). Notably, in the SeG vs. SCK group, the top five enriched metabolic pathways were the TCA cycle, glyoxylate and dicarboxylate metabolism, α-linolenic acid metabolism, carbon metabolism, and diterpenoid biosynthesis. Among these, α-linolenic acid metabolism and diterpenoid biosynthesis were significantly enriched at both the transcriptomic (*P* = 0.011 and 0.042) and metabolomic (*P* = 0.002 and 0.015) levels in the SeG vs. SCK group, suggesting they might be involved in the SeG-mediated mitigation of salt stress. Considering enrichment strength, statistical significance, pathway continuity, and functional relevance, we focused our analysis on α-linolenic acid metabolism.

We constructed a regulatory network centered on α-linolenic acid metabolism by integrating transcriptomic and metabolomic data to elucidate the potential mechanism of SeG-mediated salt stress alleviation (Fig. 5H). Metabolomic profiling revealed significant upregulation of multiple metabolites in the α-linolenic acid pathway, including the end product jasmonic acid (JA) and its precursor 12-oxo-phytodienoic acid (12-OPDA), indicating that this pathway was activated at the metabolic level. In parallel, the transcriptomic data revealed the upregulation of key biosynthetic genes such as *PLA2G*, encoding an enzyme that catalyzes membrane lipid hydrolysis to release free α-linolenic acid; and *LOX*, encoding an enzyme that initiates the oxidation of α-linolenic acid. Conversely, *AOS* expression was suppressed, but *OPR* expression remained unchanged, suggesting stage-specific or selective regulation of JA biosynthesis [34,35]. The multi-omics data reveal that SeG triggers extensive transcriptomic and metabolomic reprogramming under salt stress, encompassing antioxidant pathway responses, pronounced enrichment of α-linolenic acid metabolism, and activation of the downstream JA signaling pathway.

### 2.8 SeG-mediated modulation of the rhizosphere microbiota under salt stress

**Fig. 6:**
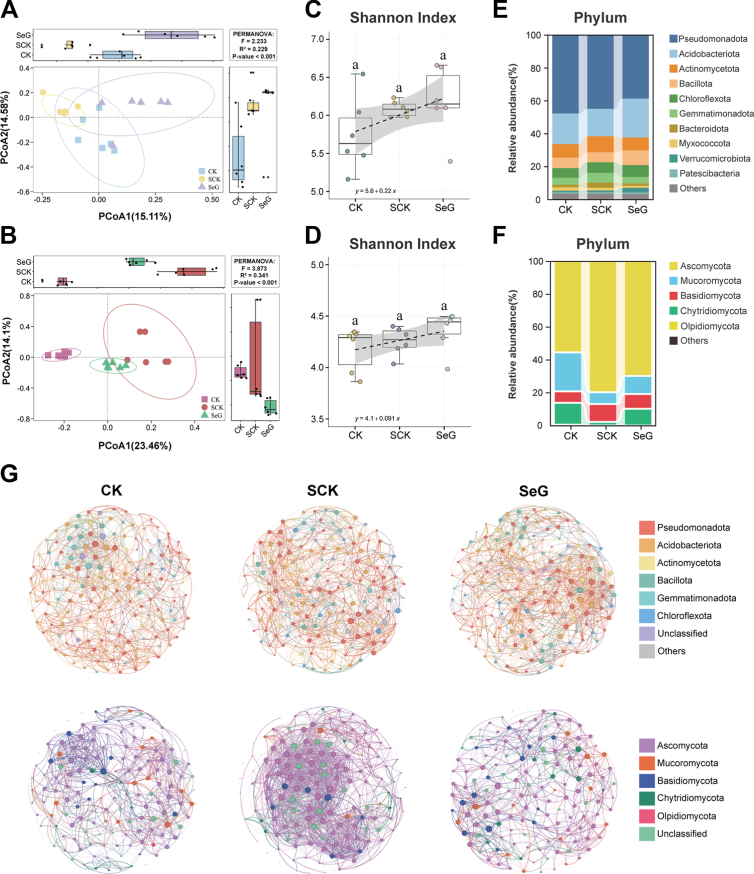
Effects of SeG treatment on the rhizosphere microbial community of soybean under salt stress. **A** Principal coordinate analysis (PCoA) of bacterial communities based on Bray-Curtis distances. **B** PCoA of fungal communities based on Bray-Curtis distances. **C** Shannon diversity index of bacteria in CK, salt control (SCK) and SeG treatments. **D** Shannon diversity index of fungi in the three treatments. **E** Relative abundance of dominant bacterial phyla across treatments. **F** Relative abundance of dominant fungal phyla across treatments. **G** Co-occurrence network analysis of bacterial and fungal communities under different treatments. Top row: bacterial networks under CK, SCK, and SeG treatments. Bottom row: fungal networks under CK, SCK, and SeG treatments. Each node represents a microbial ASV, colored by phylum-level taxonomy. Edges represent significant pairwise associations based on co-occurrence analysis. Different letters above bars indicate statistical differences among treatments (*P* < 0.05, LSD test).

Salt stress significantly altered the rhizosphere microbial community of soybean, with β-diversity analysis revealing marked changes in microbial assembly under stress in the SCK group compared to CK (Fig. 6A and B). The SeG group, however, clustered more closely with CK, suggesting that SeG treatment partially alleviated salt-induced changes in community structure. The Shannon index was higher in SeG compared to SCK, implying improved evenness and ecological richness under SeG treatment (Fig. 6C and D). At the phylum level, salt stress reduced the relative abundance of Pseudomonadota and Acidobacteriota by 5.6 % and 9.4 %, respectively, and the abundance of Actinomycetota increased by 17.0 %. SeG treatment increased the relative abundance of Bacillota by 43.9 %, mitigated the decline of Acidobacteriota, and reduced the abundance of Actinomycetota (Fig. 6E).

In the fungal community, salt stress increased the abundance of Ascomycota by 43.9 %, but SeG decreased its abundance by 12.7 %. Under SeG treatment, the abundance of Mucoromycota increased by 51.2 %, whereas that of Basidiomycota decreased (Fig. 6F). Volcano plot analysis showed that SeG treatment significantly upregulated 59 bacterial amplicon sequence variants (ASVs) and downregulated 111 ASVs; in fungi, 45 ASVs were upregulated and 30 were downregulated (Fig. S8A and B). Taxonomic annotation revealed that the upregulated ASVs included *Bacillus*, *Streptomyces*, *Penicillium*, and *Aspergillus.* Network analysis showed that salt stress disrupted microbial interactions, resulting in a decentralized bacterial co-occurrence network in the SCK group (Fig. 6G). SeG treatment restored network integrity, with increased connectivity of central nodes, suggesting that SeG facilitated the reassembly of mutualistic microbial groups. Conversely, the fungal network in SCK showed over-aggregation, which was alleviated by SeG, returning it closer to the CK state. Together, these patterns indicate that SeG helps reshape the salt-disrupted rhizosphere microbiome, restoring community balance, enriching beneficial taxa, and promoting a more resilient and functionally connected microbial network.

### 2.9 SeG-induced selection of microbes and metabolites associated with rhizosphere adaptation

To further elucidate the relationship between changes in root metabolites and rhizosphere microbial responses under SeG treatment, we first identified the top 20 upregulated DAMs (*P* < 0.05). Based on the amplicon sequencing results and the isolation of salt-tolerant microbes from rhizosphere soil (using medium containing 3 % NaCl to simulate salt stress for isolation and purification), we selected four representative microbial genera: *Bacillus*, *Streptomyces*, *Penicillium*, and *Aspergillus*. We then performed Spearman correlation analysis between the top 20 upregulated DAMs and the four microbial genera. Based on an integrated evaluation of their correlations with microbial genera, we selected three metabolites for further analysis: 3-methylglutaric acid, arbutin, and baccatin III (Fig. S9). Notably, although several metabolites exhibited higher correlation coefficients with specific microbial genera, the selection of these metabolites was not solely determined by the rank order of correlation values. Instead, we considered correlation strength and statistical significance together with their accessibility and economic feasibility, as well as suitability for potential agricultural applications. The relative abundances of these three metabolites are shown in Fig. S10A–C. Although *Streptomyces* showed no significant correlation with the top 20 enriched metabolites, it was retained due to its well-documented importance in plant stress resistance [[36], [37], [38]].

### 2.10 Functional validation of SeG-induced metabolite–microbiome interactions in the mitigation of salt stress

To evaluate the potential of both individual microbial strains and synthetic microbial communities (SynComs) to mitigate salt stress, we conducted a comparative pot experiment involving five isolates, namely *Bacillus* RSB1, *Bacillus* RSB2, *Streptomyces* RSS, *Penicillium* RSP, and *Aspergillus* RSA, along with three SynComs: SynB (RSB1, RSB2, RSS), SynF (RSP, RSA), and Syn (all five strains). These isolates were originally obtained from the rhizosphere of salt-stressed soybean plants and taxonomically identified based on their 16S rRNA or ITS sequencing. RSB1, RSB2, RSS, RSP, and RSA exhibited ≥99 % sequence similarity to *Bacillus cabrialesii*, *Bacillus velezensis*, *Streptomyces griseus*, *Penicillium citrinum*, and *Aspergillus costaricensis*, respectively (Table S2). These five isolates exhibited strong salt tolerance, as they grew normally on medium containing 8 % NaCl. Among the individual strains, RSB1, RSS, and RSP showed a certain degree of stress-alleviating efficacy, whereas RSB2 and RSA had minimal effects on plant phenotypes under salt stress (Fig. S11A–E). However, overall, the SynCom treatments outperformed the individual strain treatments. Among these, treatment with the cross-kingdom consortium Syn demonstrated the most pronounced alleviation of salt-induced phenotypic changes. The fungal consortium SynF showed significantly better performance than the single RSP and RSA treatments, suggesting that the effect of RSA might be stronger when combined with RSP. By contrast, although SynB exhibited stronger efficacy than individual bacterial strains, the poor performance of RSB2 when applied alone suggests that it contributed minimally to the overall growth-promoting capacity of the consortium.

To test this hypothesis, we constructed two additional SynCom variants excluding RSB2: SynB-1 (RSB1, RSS) and Syn-1 (RSB1, RSS, RSP, RSA). Plants treated with SynB-1 displayed plant height and aboveground biomass comparable to plants treated with SynB, with no significant differences. Similarly, Syn-1 performed on par with Syn for these growth traits (Fig. S11F–H). However, Syn-1 increased plant height by 36.4 % and aboveground biomass by 25.7 % relative to SynB-1, and Syn increased plant height by 23.1 % and biomass by 17.9 % compared with SynB. These findings confirm the notion that cross-kingdom consortia containing both bacteria and fungi promote growth more effectively than communities containing only bacteria or fungi. Moreover, the presence of RSB2 does not influence the growth-promoting effects of either the bacterial consortium or the cross-kingdom SynCom under salt stress. Consequently, the final optimized SynCom included RSB1, RSS, RSP, and RSA, omitting RSB2 (Fig. S12).

**Fig. 7:**
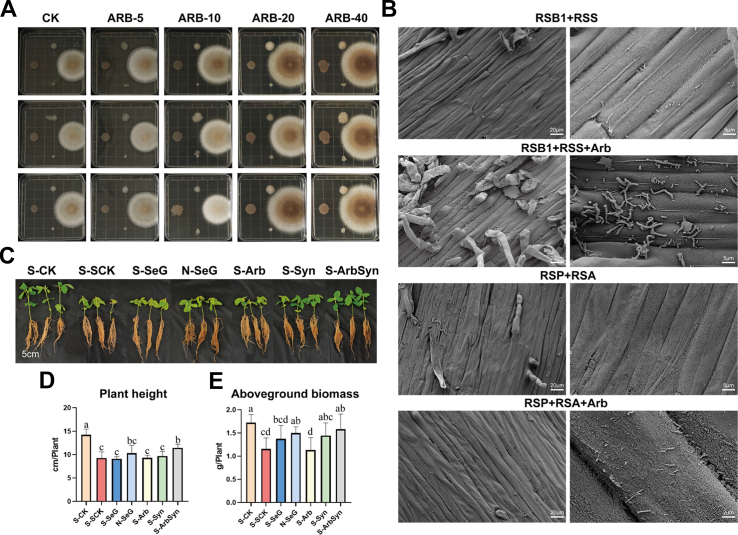
Reconstitution of SeG-induced stress alleviation via arbutin and synthetic microbial community in soybean. **A** Growth performance of Syn microbial members (RSB1, RSS, RSP, RSA) on modified nutrient agar medium supplemented with different concentrations of arbutin (5, 10, 20, and 40 mg/L) under 3 % NaCl stress. CK indicates the control without arbutin. The microbial members from top to bottom and left to right are RSP, RSS, RSB1, and RSA, respectively. **B** Observation by SEM of the effect of arbutin on the colonization of Syncom microbiota members in soybean root epidermis. **C** Phenotypic comparison of soybean plants under different treatments. S-CK: without salt stress in sterilized soil; S-SCK: salt stress in sterilized soil; S-SeG: salt stress, SeG treatment in sterilized soil; N-SeG: salt stress, SeG treatment in natural soil; S-Arb: salt stress, arbutin (40 mg/L) treatment in sterilized soil; S-Syn: salt stress, synthetic microbial consortium in sterilized soil; S-ArbSyn: salt stress, arbutin, synthetic microbial consortium in sterilized soil. **D**, **E** Plant height (D) and aboveground biomass (E) of soybean corresponding to each treatment mentioned above, *n* = 6. Different letters above bars indicate statistical differences among treatments (*P* < 0.05, LSD test).

Building on these results, we examined whether root-associated metabolites secreted by plants under salt stress enhance the growth of beneficial microbes. Specifically, we tested the effects of three representative metabolites, 3-methylglutaric acid (MGA), arbutin (Arb), and baccatin III (Bac), on the optimized SynCom members (*Bacillus* RSB1, *Streptomyces* RSS, *Penicillium* RSP, and *Aspergillus* RSA) in a simulated salt stress co-culture system. Each metabolite was applied at four concentrations, 5, 10, 20, and 40 mg/L, followed by 48 h of incubation. Arb treatment exhibited the most stable and significant growth-promoting effect. Specifically, under Arb-40 treatment, the colony diameters of all four strains surpassed those of CK (Fig. 7A). Among the SynCom members, RSP and RSA showed the most prominent increases in growth across all treatment groups, with increases of 42.5 % and 17.2 %, respectively, compared to CK (Table S3). Additionally, RSS and RSB1 exhibited growth increases of 45.7 % and 20.3 %, respectively, indicating that Arb-40 has a strong overall growth-promoting effect.

By contrast, the growth-promoting effects of MGA and Bac treatments were relatively limited and showed greater variability. For example, MGA at 20 mg/L promoted the growth of RSB1, RSS, and RSA but slightly inhibited the growth of RSP. Although Bac treatment promoted the growth of individual strains at low concentrations, the overall increases in growth were small and inconsistent. Given that Arb promoted the growth of SynCom members in *in vitro* culture experiments, we next investigated whether Arb would enhance the rhizospheric colonization capacity of these beneficial microbes, using scanning electron microscopy to observe the colonization status of the strains on the soybean root epidermis. As shown in Fig. 7B, when RSB1 and RSS were inoculated into the soybean rhizosphere without Arb (RSB1, RSS), only a few sparse microbes were observed on the root surface. By contrast, upon the addition of Arb (RSB1, RSS, Arb), a substantial number of RSB1 and RSS cells colonized the root surface, demonstrating that Arb significantly promoted their rhizospheric colonization. When soybean was inoculated with the fungal strains RSP and RSA without Arb (RSP, RSA), few fungal structures were observed. However, in the presence of Arb (RSP, RSA, Arb), numerous fungal hyphae (in the early growth stage) were visible on the root surface, indicating that Arb also promoted the colonization of these fungi in the soybean rhizosphere. Collectively, these results confirm the notion that Arb not only promotes the growth of beneficial microbes in culture medium but also facilitates their colonization in the soybean rhizosphere, which is crucial for their plant-beneficial functions.

To investigate whether the Arb–SynCom combination could recapitulate the salt-alleviating effects of SeG, we performed a functional equivalence assay. We cultivated soybean plants in sterilized or natural soil under salt stress and subjected them to one of seven treatments: S-CK (no salt stress in sterilized soil), S-SCK (salt stress in sterilized soil), S-SeG (salt stress with SeG treatment in sterilized soil), N-SeG (salt stress with SeG treatment in natural soil), S-Arb (salt stress with 40 mg/L Arb in sterilized soil), S-Syn (salt stress with SynCom in sterilized soil), and S-ArbSyn (salt stress with both Arb and SynCom in sterilized soil).

Among all treatments, N-SeG had the strongest effect on mitigating salt stress, with both plant height and aboveground biomass nearly restored to levels observed in the S-CK group (Fig. 7C–E). Remarkably, co-treatment with Arb and SynCom in sterilized soil (S-ArbSyn) achieved comparable phenotypic recovery, indicating that the ArbSyn combination functionally mimics the effect of SeG in natural soil. Specifically, S-ArbSyn increased plant height and aboveground biomass by approximately 23.4 % and 36.2 %, respectively, relative to S-SCK (*P* < 0.05), with no significant difference from N-SeG. By contrast, the individual application of Arb (S-Arb) or Syn (S-Syn) failed to reproduce the same effect. Notably, S-Arb treatment had a negligible effect on plant performance, confirming that Arb does not directly confer salt tolerance but instead acts through microbial mediation. Similarly, S-SeG treatment in sterilized soil resulted in modest improvements, underscoring the role of rhizosphere microbes in maximizing the efficacy of SeG-induced stress mitigation.

In summary, the optimized cross-kingdom SynCom outperformed individual strains and single-kingdom SynComs in mitigating salt stress in soybean. The root-secreted metabolite Arb promoted both the *in vitro* growth and rhizospheric colonization of SynCom members. Functional equivalence assays demonstrated that the Arb-SynCom combination improved the salt tolerance–enhancing effects of SeG in sterilized soil, with Arb acting indirectly via its effects on rhizosphere microbes and the efficacy of SeG also relying on these microbes.

### 2.11 Field evaluation of SeG performance in soybean and cross-species validation of its salt stress–mitigating effects in tomato and maize

Field cultivation conditions differ significantly from pot environments. Nano-fertilizers often show inconsistent performance in the field due to interactions with soil microbiomes and environmental variables. Thus, we conducted field experiments to verify the practical application value of SeG. Table S4 shows the soil physicochemical properties of the field trials.

**Fig. 8:**
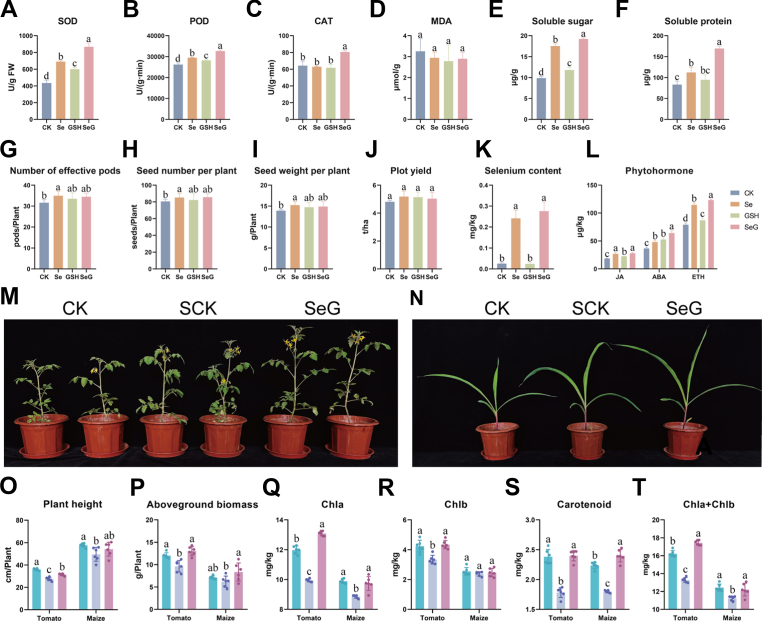
Field evaluation of SeG in soybean and pot-based salt stress validation in tomato and maize. **A**-**F** Biochemical traits measured from soybean seeds, including SOD (A), POD (B), CAT (C), MDA (D), soluble sugar (E), and soluble protein (F), *n* = 3. **G**-**J** Yield-related traits, including the number of effective pods (G), seed number per plant (H), seed weight per plant (I), and plot yield (J), *n* = 5. **K** Selenium content quantified from soybean seeds, *n* = 6. **L** Phytohormone levels quantified from soybean seeds, *n* = 3. **M**, **N** Phenotypic images of tomato (M) and maize (N) under different treatments. **O**-**T** Physiological indicators of tomato and maize under the different treatments, including plant height (O), aboveground biomass (P), chlorophyll *a* (Q), chlorophyll *b* (R), carotenoids (S), and total chlorophyll (T), *n* = 6. Different letters above bars indicate statistical differences among treatments (*P* < 0.05, LSD test).

SeG significantly promoted metabolism, yield, and selenium accumulation in soybean plants grown in the field. SeG treatment significantly increased antioxidant enzyme activities in soybean seeds, with SOD, POD, and CAT activities increasing by 99.8 %, 24.7 %, and 25.7 %, respectively, compared to CK (Fig. 8A–C). SeG also reduced MDA content by 10.98 % and significantly increased soluble sugar and soluble protein contents, which were 94.6 % and 103.6 % higher than CK, respectively (Fig. 8D–F). SeG also promoted yield formation. The number of effective pods, seeds per plant, seed weight per plant, and plot yield increased by 9.1 %, 6.2 %, 7.1 %, and 4.7 %, respectively, compared to CK (Fig. 8G–J). Although these increases did not reach statistical significance, they still demonstrated stable yield-increasing potential. In addition, SeG treatment markedly increased the selenium content in soybean seeds, reaching 8.8-fold that of CK (Fig. 8K). Notably, the selenium content remained within the safe consumption range, suggesting that SeG improved yield and enhanced the nutritional quality of soybean seeds. SeG also significantly increased the levels of stress-related plant hormones in soybean seeds, with JA, abscisic acid, and ethylene concentrations 51.0 %, 74.4 %, and 56.2 % higher, respectively, than those in CK (Fig. 8L).

To assess the potential cross-species conservation of SeG-mediated regulation, we conducted salt stress pot experiments using tomato (*Solanum lycopersicum*) and maize (*Zea mays*; Fig. 8M and N). Salt stress significantly suppressed plant growth in both tomato and maize: compared to CK, tomato exhibited a 23.6 % reduction in plant height and a 20.0 % reduction in aboveground biomass, whereas maize showed corresponding decreases of 13.9 % and 13.0 %, respectively (Fig. 8O and P). SeG treatment alleviated these inhibitory effects. Tomato plant height increased by 14.2 % relative to SCK, whereas maize plant height increased by 9.2 %. Improvements in aboveground biomass were even more pronounced: aboveground biomass increased by 35.5 % in tomato and 34.8 % in maize relative to SCK. Salt stress markedly decreased photosynthetic pigment levels in both crops, whereas SeG treatment provided clear protective effects on pigment accumulation (Fig. 8Q-T). Chlorophyll *a*, chlorophyll *b*, carotenoid, and total chlorophyll levels in tomato increased by 31.7 %, 30.2 %, 34.6 %, and 31.3 % relative to SCK. In maize, chlorophyll *a*, chlorophyll *b*, carotenoid, and total chlorophyll levels increased by 9.9 %, 3.7 %, 33.6 %, and 8.6 %, respectively, relative to SCK.

Collectively, our field data demonstrate that SeG treatment enhances soybean yield, improves seed nutritional traits, and strengthens plant defense responses, including increases in antioxidant enzyme activities and the levels of stress-related phytohormones. Cross-species validation showed that SeG confers robust protection against salt stress in both tomato and maize, indicating that its regulatory effects are conserved across species.

## 3 Discussion

Soil salinization poses a critical challenge to global agricultural sustainability, affecting crop productivity and disrupting the ecological equilibrium. To address this issue, we examined the effects of treating salt-stressed soybean plants with SeNPs@CS nanomaterials. SeNPs@CS exhibit superior dispersibility and stability compared to conventional SeNPs, enabling sustained selenium release within plant tissues [29,39]. The foliar application of SeNPs@CS in combination with GSH (SeG) on soybean promoted physiological processes such as ROS scavenging and helped maintain the photosynthetic apparatus under salt stress. Concurrently, this treatment activated the JA pathway, driving the accumulation of Arb and its release as a signaling molecule into the rhizosphere. In turn, Arb recruited plant-growth-promoting microorganisms that enhance salt tolerance, thereby alleviating salt stress in soybean.

SeNPs with specific ligand modifications exert their effects through receptor binding [40], but this mechanism remains unreported in plants. In this study, our multi-omics data indicate that the JA pathway was activated following SeNPs@CS treatment; however, the upstream events remain unresolved. Based on current knowledge, we suggest that SeNPs@CS interact with yet-to-be-identified membrane-associated receptors and trigger intracellular calcium signaling and MAPK cascades, thereby enhancing the expression of JA biosynthetic genes such as *LOX* and *AOS* [41,42]. This proposed model places our observations within existing signaling paradigms, but it remains speculative. Further work will be needed to identify the specific receptor(s) and signaling components involved, including studies focused on receptor characterization and pathway-resolved genetic or biochemical analyses. GSH regulates the redox status in plants by directly scavenging stress-induced ROS [10,43]; this reduces damage to key signaling proteins in the JA pathway (e.g., the JA receptor COI1), ensuring efficient signal transduction. GSH and SeNPs@CS jointly improve soybean salt tolerance through complementary functions: GSH contributes to the maintenance of cellular redox homeostasis, whereas SeNPs@CS promotes signal transduction, stabilizes membrane lipids via the α-linolenic acid pathway, and enhances the activation of JA-mediated defense responses. This mechanism overcomes the limitations of treating plants with single substances that only have antioxidant or signal regulation functions, thereby achieving efficient regulation of plant salt tolerance.

SeG treatment activates multiple metabolic pathways in soybean and significantly promotes the accumulation of Arb. This plant secondary metabolite plays a key role in plant salt tolerance through a dual mechanism: its hydroquinone group scavenges salt stress-induced ROS via electron transfer, exerting antioxidant effects; and its glucosyl moiety enhances its affinity for the membrane surface, allowing the hydroquinone group to be inserted into the lipid bilayer, reducing Na^+^-mediated damage to membrane proteins and stabilizing cell membrane structure [44,45]. However, the role of Arb is not limited to direct regulation of plant physiological processes. Our findings reveal the novel role of Arb as a rhizospheric chemical signaling molecule during plant salt tolerance, in addition to its physiological functions. Under salt stress, SeG-treated soybean secretes Arb into the rhizosphere as a selective chemical signal, regulating the behavior and distribution of salt-tolerant plant-growth-promoting microorganisms to enhance the enrichment of beneficial microbes such as *Bacillus*, *Streptomyces*, *Penicillium*, and *Aspergillus*.

Evidence from non-plant systems supports a potential role for Arb in modulating microbial communities. For example, in murine models, Arb reshapes gut microbial communities by enriching the accumulation beneficial bacteria and short-chain-fatty-acid-producing taxa while suppressing pathogen growth [46,47]. These cross-system findings suggest that Arb can modulate microbial assemblages, supporting its role as a rhizospheric regulatory molecule in plants. Furthermore, a recent soil-based study provided mechanistic insights into the metabolic fate of Arb in plant–microbe symbiotic environments [48]. In soils used for lettuce (*Lactuca sativa*) cultivation, Arb was shown to be hydrolyzed by microbial β-glucosidase into glucose and hydroquinone (H_2_Q). Glucose serves as an accessible carbon source fueling microbial metabolism, whereas H_2_Q functions as an electron donor that mitigates oxidative stress and mobilizes iron, creating a rhizospheric environment favorable for microbial proliferation. Importantly, the beneficial genera enriched in the current study (*Bacillus*, *Streptomyces*, *Penicillium*, and *Aspergillus*) produce β-glucosidase [49], equipping them with a competitive advantage to use Arb as both a nutrient substrate and a stress-alleviating cofactor. This Arb-mediated metabolic compatibility could explain their selective enrichment in the soybean rhizosphere and provides a basis for understanding how these recruited microbes subsequently contribute to plant salt tolerance.

These enriched microorganisms demonstrate favorable or even normal growth under salt stress conditions and help alleviate salt stress in soybean. For example, various *Bacillus* strains enhance plant salt tolerance by modulating phytohormone biosynthesis, promoting the accumulation of osmoprotectants (e.g., proline), activating antioxidant systems, and maintaining Na^+^/K^+^ homeostasis [50,51]. In addition, *Streptomyces* species such as *Streptomyces lasalocidi* JCM 3373ᵀ secrete indole-3-carboxaldehyde (ICA1d), stimulate POD and SOD activities, increase proline and soluble sugar levels, and upregulate genes involved in root growth and stress responses, thereby alleviating salt-induced damage in soybean [36].

Fungal taxa also participate in plant acclimation to salt stress through distinct mechanisms. For instance, *Penicillium olsonii* enhances salt tolerance in tobacco (*Nicotiana tabacum*) by secreting metabolites that upregulate genes involved in auxin and brassinosteroid biosynthesis, promoting root development, reducing Na^+^ uptake, and enhancing K^+^ acquisition, thereby maintaining ionic balance and sustaining root vitality [52]. *Aspergillus aculeatus* secretes organic acids and siderophores under salt stress, improving nutrient uptake and alleviating oxidative damage and metabolic disturbance, ultimately enhancing growth performance, photosynthetic efficiency, and nutritional quality in ryegrass (*Lolium perenne*) [53].

Notably, among the microorganisms recruited by Arb, the fungi-bacteria cross-kingdom synthetic community outperformed single bacterial or fungal synthetic communities in enhancing salt tolerance in soybean. This superiority likely arises from functional complementarity, metabolic synergy, and stable stress resistance among taxa, thereby comprehensively meeting the complex rhizospheric demands of soybean under salt stress. Although single bacterial communities excel in secreting osmotic regulators (e.g., proline, betaine) or activating rhizospheric phosphorus via phytase, they lack the capacity to improve soil physical structure or adsorb salt ions on a large scale [54,55]. Even though single fungal communities extend soil exploration through mycelial networks, adsorb excessive Na^+^, and secrete polysaccharides to optimize soil aggregate structure, they fail to rapidly supply available nutrients or synthesize abundant osmotic regulators [[56], [57], [58]]. These limitations render single synthetic communities incapable of comprehensively addressing the multiple challenges of salt ion toxicity, nutrient imbalance, and oxidative stress under salt stress, leading to unstable salt tolerance effects. In cross-kingdom synthetic communities, however, Na^+^ adsorption by fungal mycelia creates conditions for bacterial survival in saline environments, while the bacteria provide carbon sources for fungi through organic matter decomposition. This stress-resistant cross-kingdom community structure, with mutual buffering and supporting effects, helps ensure the sustained stability of community structure and function.

Building on these findings, we investigated how the metabolite Arb and cross-kingdom synthetic microbial communities help alleviate salt stress in soybean. The single application of Arb or cross-kingdom synthetic communities in sterilized soil failed to replicate the effect of SeG treatment on mitigating salt stress in natural soils. By contrast, their combined application in sterilized soil alleviated salt stress with an effect comparable to that of SeG treatment in natural soil. Therefore, Arb does not directly enhance salt tolerance in soybean but exerts this effect by modulating the composition and function of rhizospheric microbial communities. This observation is consistent with the finding that plant-secreted metabolites typically enhance plant stress resistance by regulating rhizospheric microbial communities rather than exerting direct physiological effects on plants themselves [21,59,60]. However, the effect of single application of cross-kingdom synthetic communities was also limited. Although cross-kingdom synthetic communities regulate the rhizospheric environment and enhance plant adaptability to stress to some extent, these effects must be maximized through synergistic interactions with plant metabolites. When Arb serves as a rhizospheric signaling molecule, it activates the functions of cross-kingdom synthetic communities, promotes their enrichment in the rhizosphere, thereby amplifying their effects on alleviating salt stress. This collaborative mechanism, where plant metabolites act as signals for microorganisms, helps explain how SeG treatment mitigates salt stress in soybean.

However, this study had several limitations. First, although we validated the efficacy and reproducibility of SeG in the field in non-saline soil, its performance across different soil types and climatic conditions and its long-term dynamic effects on crop yield and soil microbial diversity remain to be fully evaluated. Second, although Arb promoted the growth of beneficial microbes in the soybean rhizosphere, whether it induces the expression of microbial chemotaxis-related genes (e.g., *cheA*, *flaB*) to promote flagellar movement for directional aggregation toward the soybean rhizosphere remains to be verified. An in-depth understanding of these mechanisms would provide more precise theoretical support for optimizing SeG treatment in saline soils. Third, our multi-omics analysis suggested that the JA pathway helps mediate SeG-induced salt tolerance, but we did not perform genetic validation using JA biosynthesis or signaling mutants. Further studies employing genetic approaches (e.g., CRISPR/Cas9-generated mutants) are needed to directly confirm the causal role of JA in this process and to further strengthen the mechanistic framework of SeG action.

**Fig. 9:**
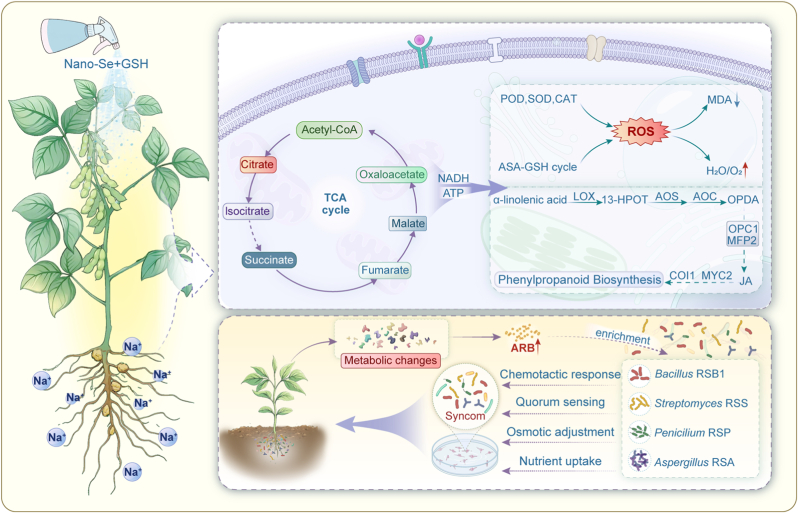
Schematic model of SeG-mediated salt tolerance in soybean. SeG alleviates oxidative damage by enhancing antioxidant enzyme activities and the ASA-GSH cycle, reducing ROS and lipid peroxidation. It also triggers metabolic reprogramming, activating α-linolenic acid pathway, upregulating key genes (e.g., *LOX*, *OPC1*), and promoting JA accumulation. These shifts modify root exudates, particularly enriching Arb, which acts as a signaling molecule to recruit a Syncom (*Bacillus* RSB1, *Streptomyces* RSS, *Penicillium* RSP, *Aspergillus* RSA). The Syncom likely promotes plant growth and salt tolerance via cooperative colonization, quorum sensing, nutrient uptake, osmotic regulation, and other beneficial physiological effects.

In conclusion, our study unveiled the synergistic effect of combined SeNPs@CS and GSH application in mitigating salt stress. SeG enhances the soybean antioxidant system, activates the JA signaling pathway in response to salt stress, and regulates the rhizospheric microbial community through the release of the rhizospheric metabolite Arb, thereby significantly improving salt tolerance in soybean (Fig. 9). This finding supports the notion that plant metabolites enhance plant stress resistance by regulating the microbial community and that the combined application of SeNPs, GSH, and plant metabolites could provide an efficient, sustainable solution for agricultural production in saline soils. By integrating the advantages of plant, microbial, and nanotechnology approaches, this strategy offers a practical, innovative pathway to address the increasingly severe global issue of soil salinization.

## 4 Materials and methods

### 4.1 Synthesis of SeNPs@CS

SeNPs@CS were synthesized by chemical reduction as described by Bai [61], with minor modifications. In brief, 0.5 g chitosan was accurately weighed and added to 50 mL of a 1 % acetic acid solution. The mixture was stirred at 600 rpm at room temperature for 6 h to ensure complete dissolution of chitosan. Then, 0.111 g of selenium dioxide (SeO_2_) was weighed and dissolved in 50 mL of pure water to prepare a 20 mM H_2_SeO_3_ solution. Next, 25 mL of the H_2_SeO_3_ solution was slowly added dropwise to the chitosan solution. After the addition, the mixture was further stirred for 30 min. Subsequently, 10 mL of a 2 % ascorbic acid solution was added dropwise to the mixed solution, followed by 3 h of continuous stirring. A color change from colorless to red indicated the formation of SeNPs. After the reaction, the solution was transferred into a dialysis bag and dialyzed in pure water for dialysis. The dialysis (with dialysate replacement every 6 h) lasted 24 h to remove unreacted ascorbic acid and by-products.

### 4.2 Morphological characterization of SeNPs@CS

A silicon wafer is selected, ensuring the smooth side faces upward, and is then adhered to the carbon conductive tape. A small amount of the diluted SeNPs solution is drawn using a pipette and dropped onto the silicon wafer. Once the sample is fully dry, it can be observed under a scanning electron microscope (SU8020, HITACHI, Japan). The microspheres are sputter-coated with platinum under vacuum conditions, and images are taken at an acceleration voltage of 3–5 kV [[62], [63], [64]]. An in-built energy-dispersive X-ray spectrometer (EMAX mics2, HORIBA, Japan) is used for simultaneous elemental analysis during SEM observation.

The particle size of the SeNPs@CS is analyzed using a particle size analyzer (Zetasizer Nano ZS90, Malvern, UK). The instrument is preheated for 30 min before use. The diluted sample solution is ultrasonically dispersed for 5 min, then 1 mL of the upper-layer solution is taken and slowly added to the sample cell. The particle size test program is then started, with water selected as the solvent, and the system is preheated at 25 °C for 30 s before measurement. Each sample is measured in triplicate.

### 4.3 Preparation of SeNPs@CS-FITC

To prepare fluorescently labeled SeNPs@CS (SeNPs@CS-FITC), a 0.2 mg/mL FITC solution in methanol was prepared and stored in the dark. Then, 10 mL of the FITC solution was slowly added dropwise to 10 mL of SeNPs@CS solution, which had been pre-diluted to 100 mg/L. The mixture was sonicated in a water bath for 30 min and incubated in the dark for 2 h to promote covalent binding between FITC and the amino groups on the chitosan shell. After the reaction, the mixture was transferred into a dialysis bag (MWCO 5 kDa) and dialyzed against deionized water at 4 °C in the dark, with the dialysate refreshed every 6 h. Dialysis was continued until no fluorescence signal was detected in the external solution, indicating the complete removal of unbound FITC. The resulting SeNPs@CS-FITC solution was diluted to 10 mg/L and stored at 4 °C in the dark for subsequent fluorescence tracing experiments.

### 4.4 Foliar treatment and confocal microscopy of SeNPs@CS-FITC

Soybean seedlings at the trifoliate leaf stage were selected and treated by foliar spraying with SeNPs@CS-FITC. Leaf and root samples were collected at 2 h and 6 h post-treatment, respectively, under dark conditions. The samples were immediately fixed in 2.5 % glutaraldehyde solution at 4 °C overnight.

Fresh root segments (0.5 cm) were embedded in Tissue Freezing Medium and snap-frozen on the freezing stage of a cryostat (CM1950, Leica, Germany) for 10 min. The root segments were then vertically embedded into a pre-frozen base layer, covered with additional Tissue Freezing Medium, and frozen again at −25 °C for 20 min. Re-embedding and freezing were performed if necessary to stabilize the sectioning orientation. Once fully frozen, 50 μm-thick sections were prepared and mounted onto glass slides. Approximately 30 μL of deionized water was added to each section before covering with a coverslip. Fluorescence imaging was performed using a confocal laser scanning microscope (FV3000, Olympus, Japan). According to the spectral characteristics of FITC, the excitation and emission wavelengths were set at 488 nm and 517 nm, respectively.

### 4.5 Experiment design

This experiment was conducted at the Guangdong Subcenter of National Center for Soybean Imporvement, South China Agricultural University. The soybean variety selected is Huaxia No.9, which is provided by the Guangdong Subcenter of National Center for Soybean Improvement. First, soybean seeds were placed in seedling pots filled with vermiculite. Once the cotyledons had emerged, the seedlings were transplanted into flowerpots containing 1.2 kg of soil, with two seedlings per pot. The light cycle was set to 13 h of light and 10 h of darkness. The cultivation temperature was maintained at 27 ± 0.5 °C, with a relative humidity of 65 %. The experiment was divided into three parts.

The first part aimed to determine the optimal foliar application concentrations of SeNPs and GSH. SeNPs concentrations were set at 0, 5, 10, 20, and 40 mg/L, while GSH concentrations were 0, 10, 25, 50, 100 and 200 mg/L. The second part included four treatment groups: the control group (CK), 10 mg/L SeNPs treatment (Se), 25 mg/L GSH treatment (GSH), and the combined 10 mg/L SeNPs and 25 mg/L GSH treatment (SeG). CK plants were sprayed with pure water. Foliar spraying was initiated when the trifoliolate leaves were fully expanded and repeated every five days. On the fifth day after the fourth spraying, growth parameters, photosynthetic indicators, and antioxidant enzyme activities were measured. The third part included five treatment groups: CK, salt-stressed control (SCK), Se, GSH, and SeG. The treatment protocol was consistent with that in the second part, except that during the second foliar spraying, 400 mL of 200 mM NaCl solution was applied to all groups except CK to induce salt stress. On the fifth day after the fourth spraying, leaf, root, and rhizosphere soil samples were collected. The leaf samples were immediately used to assess photosynthetic performance and measure antioxidant enzyme activities, while the rest were snap-frozen in liquid nitrogen and stored at −80 °C. The entire sampling process was completed within 15 min. Rhizosphere soil samples were used for microbial diversity analysis, while root tissues were used for metabolomic and transcriptomic analyses.

### 4.6 Measurement of photosynthetic parameters

The steps for extracting photosynthetic pigments are as follows: Wash fresh soybean leaves, blot dry with absorbent paper, remove the veins, and cut the leaves into small pieces. Weigh 0.1 g of the chopped fresh sample, add 10 mL of 95 % ethanol, shake well, and extract in the dark for 24 h. Take 1 mL of the supernatant, dilute it with 3 mL of 95 % ethanol, and measure the absorbance at wavelengths of 665 nm, 649 nm, and 470 nm using a spectrophotometer (UV-1800, Japan) [65]. Use 95 % ethanol as the blank control for calibration.

### 4.7 Measurement of antioxidant indexes

The determination of antioxidant activity was performed using the kit method. For the determination of superoxide dismutase (SOD), peroxidase (POD), malondialdehyde (MDA), catalase (CAT), phenylalanine ammonia-lyase (PAL), ascorbate peroxidase (APX), reduced glutathione (GSH), and total antioxidant capacity, the following kits from Genepioneer Biotechnologies Co., Ltd. (Nanjing, China) were used: Superoxide Dismutase Kit (JC0101-M), Peroxidase Kit (JC0102-S), Malondialdehyde Assay Kit (JC0202-M), Phenylalanine Ammonia-lyase Kit (JC0114-M), Catalase Assay Kit (JC0103-S), Ascorbate Peroxidase (APX) Kit (JC1203-S), Reduced Glutathione (GSH) Kit (JC1103-M), and Total Antioxidant Capacity (ABTS Method) Kit (JC0119-M).

The determination of glutathione peroxidase and monodehydroascorbate reductase was performed using the Glutathione Peroxidase Test Kit (GPX-2-W) and Monodehydroascorbate Reductase Test Kit (MDHAR-2-W) from Suzhou Keming Biotechnology Co., Ltd. (Jiangsu, China).

### 4.8 Transcriptome analysis

The total RNA was extracted using the TRIzol® reagent kit (Invitrogen) following the manufacturer's protocol, and genomic DNA was removed using DNase I (TaKara). The RNA quality and concentration were assessed using the Agilent 2100 Bioanalyzer (Agilent Technologies) and NanoDrop 2000 (NanoDrop Technologies), ensuring the samples meet the required quality standards. 1 μg of high-quality RNA was used to construct cDNA libraries. The library preparation process included polyA selection of mRNA, fragmentation, reverse transcription to synthesize cDNA, end repair, A-tailing, and adapter ligation. Library size selection was performed by using a 2 % low-range Ultra agarose gel, followed by PCR amplification using Phusion DNA polymerase (NEB). The final library was quantified using a TBS380 and sequenced on the Illumina platform with 150 bp paired-end reads. Raw sequencing data were processed with Trimmomatic for quality control and adapter removal. Specifically, low-quality sequences and those shorter than 75 bp were excluded. High-quality reads after trimming were aligned to the reference genome using Hisat2, and quality assessment was performed using Qualimap. Gene expression levels were quantified using featureCounts, and differentially expressed genes (DEGs) were identified with the edgeR R package, using the criteria of |log_2_ fold change| ≥ 2 and FDR <0.05. Gene Ontology (GO) enrichment and Kyoto Encyclopedia of Genes and Genomes (KEGG) pathway analyses were performed using Goatools and KOBAS, respectively. Alternative splicing events were analyzed using MATS, and SNP detection was carried out using Samtools and GATK. Data analysis was conducted based on these procedures, aiming to accurately identify and annotate each gene and functional module.

### 4.9 Metabolomic analysis

After grinding the samples with liquid nitrogen, a pre-chilled solution containing 80 % methanol and 0.1 % formic acid was added, followed by thorough vortexing. The samples were then placed on ice for a 5-min incubation, after which they were centrifuged at 15,000 rpm and 4 °C for 5 min. A portion of the supernatant was diluted using LC-MS grade water to adjust the final methanol concentration to 53 %. The samples were transferred to a new Eppendorf tube and centrifuged again at 15,000 *g* under 4 °C for 10 min. The obtained supernatant was subsequently injected into the LC-MS/MS system for analysis. The analysis was performed using a Vanquish UHPLC system (Thermo Fisher, Germany) coupled with an Orbitrap Q Exactive™ HF mass spectrometer (Thermo Fisher, Germany) at Biozeron Co., Ltd. (Shanghai, China). The samples were injected onto a Hypersil Gold column (100 × 2.1 mm, 1.9 μm) and eluted with a 17-min linear gradient at a flow rate of 0.2 mL/min. In positive ion mode, mobile phase A consisted of water containing 0.1 % formic acid, and mobile phase B was methanol. In negative ion mode, mobile phase A was 5 mM ammonium acetate (pH 9.0), and mobile phase B was methanol. The elution gradient was as follows: 0–1.5 min, 2 % B; 1.5–13.5 min, B increased linearly from 2 % to 100 %; 13.5–14 min, 100 % B; 14–14.1 min, B decreased from 100 % to 2 %; 14.1–17 min, 2 % B. The Q Exactive™ HF mass spectrometer was operated in both positive and negative ion modes with a spray voltage of 3.2 kV, a capillary temperature of 320 °C, a sheath gas flow rate of 40 arb, and an auxiliary gas flow rate of 10 arb.

The raw data files produced by UHPLC-MS/MS were processed with Compound Discoverer 3.1 (Thermo Fisher Scientific) to conduct peak alignment, peak picking, and other analytical procedures. Metabolite molecular formulas were identified by considering additive ions, molecular ion peaks, and fragment ions. Both qualitative and relative quantitative analyses were carried out through matching with the MassList database, using the following parameters: a retention time tolerance of 0.2 min, mass tolerance of 5 ppm, signal intensity tolerance of 30 %, signal-to-noise ratio of 3, and a minimum intensity of 100,000. Once peak intensities were normalized to the total spectral intensity, the data were matched against the mzCloud (https://www.mzcloud.org/) and mzVault databases. Statistical analyses were performed using R software (version 3.4.3), with the area normalization method applied for data that exhibited a normal distribution.

### 4.10 Microbial genome sequencing

Microbial DNA was extracted from rhizosphere soil samples using the E. Z.N.A.® Soil DNA Kit (Omega Bio-Tek, Norcross, GA, U.S.) according to the manufacturer's protocol. The V3–V4 region of the bacterial 16S rRNA gene was amplified using primers 341F (5' - CCTAYGGGRBGCASCAG - 3′) and 806R (5' - GGACTACNNGGGTATCTAAT - 3′). Fungal DNA was analyzed by amplifying the ITS1F-ITS2R region using primers ITS1 (5' - CTTGGTCATTTAGAGGAAGTAA - 3′) and ITS2 (5' - GCTGCGTTCTTCATCGATGC - 3′) [66]. PCR reactions for each sample were performed in triplicate, with a 20 μL reaction mixture consisting of 4 μL of 5 × FastPfu Buffer, 2 μL of 2.5 mM dNTPs, 0.8 μL of each primer (5 μM), 0.4 μL of FastPfu Polymerase, and 10 ng of template DNA. The PCR program consisted of an initial denaturation at 94 °C for 2 min, followed by 32 cycles of denaturation at 94 °C for 30 s, annealing at 55 °C for 30 s, and extension at 72 °C for 50 s. A final extension was performed at 72 °C for 5 min [67]. Following amplification, the PCR products were extracted from a 2 % agarose gel and purified with the AxyPrep DNA Gel Extraction Kit (Axygen Biosciences, Union City, CA, U.S.) following the manufacturer's protocols. The purified PCR products were quantified using a Qubit® 3.0 Fluorometer. Twenty-four amplicons with different barcodes were pooled in equal amounts. The pooled DNA was used to construct an Illumina paired-end library following Illumina's genomic DNA library preparation protocol. The amplicon library was sequenced (2 × 250) on an Illumina MiSeq platform (Shanghai BIOZERON Co., Ltd.) following standard procedures.

### 4.11 Isolation and identification of cultivable microorganisms

One gram of rhizosphere soil from soybean was transferred into a 50 mL sterile centrifuge tube containing 9 mL of sterile water. The mixture was shaken at 200 rpm and 37 °C for 30 min to prepare a soil microbial suspension. The suspension was serially diluted, and aliquots from the 10^−3^, 10^−4^, and 10^−5^ dilutions were spread onto LB agar (for bacteria) and PDA agar (for fungi) plates [68]. Plates were incubated at 30 °C, with bacteria grown for 1–2 days and fungi for 1–7 days. Individual colonies were isolated and streaked repeatedly on the corresponding medium to obtain pure cultures.

Genomic DNA was extracted from the purified strains, and the 16S rRNA gene (for bacteria) or ITS region (for fungi) was amplified by PCR. The PCR products were verified by agarose gel electrophoresis and sent for sequencing (conducted by Shanghai Majorbio Bio-Pharm Technology Co., Ltd). The raw sequences were assembled and dereplicated using standard bioinformatic tools. Taxonomic classification was performed using NCBI BLAST, and representative strains were selected by comparing their sequences to ASV representative sequences obtained from high-throughput sequencing in this study (identity ≥95 %). A phylogenetic tree was constructed using the neighbor-joining method implemented in MEGA 12.0 [69,70].

### 4.12 Screening of salt-tolerant microbial strains

The purified bacterial and fungal isolates were inoculated onto LB and PDA agar plates containing 3 %, 5 %, and 8 % NaCl. Bacteria were incubated at 37 °C, and fungi at 28 °C. Strains capable of growing under these saline conditions were identified as salt-tolerant for further analysis.

### 4.13 Screening of single strains and synthetic microbial communities for soybean salt stress alleviation

This experiment included nine treatment groups, and all conducted in sterilized soil with a soil weight of 1.2 kg per pot. The treatments consisted of a salt stress control (SCK); five single-strain treatments using *Bacillus* RSB1, *Bacillus* RSB2, *Streptomyces* RSS, *Penicillium* RSP, and *Aspergillu*s RSA; a bacterial consortium (RSB1, RSB2, RSS); a fungal consortium (RSP, RSA); and a combined bacterial-fungal consortium (RSB1, RSB2, RSS, RSP, RSA).

When the first trifoliate leaves of soybean seedlings were fully expanded, each group was treated by root drenching with 40 mL of the corresponding microbial suspension. After 48 h of inoculation, all groups were subjected to salt stress by root drenching with 400 mL of 200 mmol L^−1^ NaCl solution. Once clear phenotypic differences emerged, growth-related parameters were immediately measured for each group.

### 4.14 Plate verification of the growth-promoting effects of metabolites in an artificial microbiome background

A solid plate assay was conducted to evaluate the effects of specific metabolites on microbial growth under a simulated co-culture environment. The culture medium adopted the basic formula of Nutrient Agar (NA), with an additional 10 g/L glucose to promote fungal growth, and 3 % NaCl was uniformly added to simulate a salt-stress environment. There were three metabolite treatments: arbutin, 3-methylglutaric acid, and baccatin III. Each metabolite was set with five concentration gradients: 0, 5, 10, 20, and 40 mg/L. Considering the thermal instability of arbutin and baccatin III, all metabolite solutions were subjected to sterilization treatment using 0.22 μm filters. Subsequently, 100 μL of the metabolite solution was added to each plate, evenly spread on the surface, and after drying, microbial inoculation was carried out.

Each plate was inoculated with 4 microbial strains: RSB1, RSS, RSP, and RSA. The spot inoculation method was used, with an inoculum size of 2 μL per strain, which were spotted in the four directions of up, down, left, and right of the plate respectively to simulate the symbiotic environment of the artificial microbiome without setting partitions. During the culture process, the growth of the colonies was observed regularly, and indicators such as changes in colony diameter and growth rate were recorded.

### 4.15 Evaluation of the combined effects of SynCom and metabolite in a pot assay

A pot experiment was carried out to assess the combined effects of the synthetic microbial community (SynCom) and the selected metabolite on the salt stress tolerance of soybean. Six treatment groups were included: (1) Sterilized soil under salt stress (control); (2) Natural soil under salt stress with SeG treatment; (3) Sterilized soil under salt stress with SeG treatment; (4) Sterilized soil under salt stress treated with Syncom; (5) Sterilized soil under salt stress with arbutin treatment; (6) Sterilized soil under salt stress treated with both SynCom and arbutin. The SynCom was composed of four microbial strains: *Bacillus* RSB1, *Streptomyces* RSS, *Penicillium* RSP, and *Aspergillus* RSA. The metabolite treatment involved 40 mg/L arbutin.

Soybean seeds were surface-sterilized with 70 % ethanol followed by 2.5 % sodium hypochlorite prior to germination [71]. When the first true leaves of soybean seedlings were fully expanded, foliar application of SeG, root drenching of microbial suspensions, and addition of the metabolite were performed according to treatment. After 24 h, all groups were subjected to salt stress by root drenching with 200 mL of 200 mmol L^−1^ NaCl solution. Plant phenotypes were monitored regularly, and growth-related parameters were measured once clear differences emerged among treatments.

### 4.16 Field trial

The field experiment was conducted at an experimental field in Qiqihar, Heilongjiang Province, China. The soybean cultivar Qinong 60 was used in this study. Sowing was carried out in mid-August 2025, and standard agronomic management practices, including thinning, weed control, fertilization, and irrigation, were applied uniformly throughout the growing season. Plants were spaced at 50 cm between rows and 10 cm within rows. The experiment was arranged in a randomized complete block design, with each treatment replicated in five independent blocks. Foliar application of SeG was performed twice, once at the flowering stage and once at the pod-setting stage. At maturity, plot yield was measured, yield components were determined, and physiological parameters were analyzed following standard procedures.

### 4.17 Cross-species validation experiment

To evaluate whether the SeG-mediated stress-alleviating effect is conserved across plant species, additional pot experiments were conducted using tomato and maize. Experimental procedures were carried out with reference to Section 4.5, except that soybean was replaced by tomato or maize.

### 4.18 Statistical analysis

Statistical analyses were performed with SPSS 26.0 (IBM SPSS, Armonk, New York, USA). Student's *t*-tests and one-way analysis of variance (ANOVA) were performed to assess differences among treatment groups, with significance defined at *P* < 0.05. Data visualization was carried out using GraphPad Prism 8.0.1 (GraphPad Software, USA), R (v4.4.2), and the online platform OmicShare (https://www.omicshare.com). Microbial co-occurrence networks were constructed and visualized using Gephi (v0.8.2) as described previously [72,73].

## CRediT authorship contribution statement

**Yanxi Chen:** Writing – original draft, Methodology, Investigation, Formal analysis, Data curation. **Shuai Zhao:** Writing – review & editing, Supervision, Conceptualization. **Xuanxuan Ma:** Methodology, Investigation. **Li Ling:** Investigation. **Pengxin Du:** Investigation. **Xu Liu:** Investigation. **Yuhao Yao:** Investigation. **Qibin Ma:** Writing – review & editing, Supervision. **Yanbo Cheng:** Writing – review & editing, Supervision. **Yingxiang Wang:** Writing – review & editing, Supervision. **Jian Wei:** Writing – review & editing, Supervision. **Hai Nian:** Writing – review & editing, Supervision, Conceptualization. **Tengxiang Lian:** Writing – review & editing, Writing – original draft, Supervision, Conceptualization.

## Declaration of competing interest

The authors declare that they have no competing interests.

## Data Availability

The amplicon raw sequencing data and RNA-Seq data for all samples used in this study have been deposited in the National Center for Biotechnology Information (NCBI) database. Specifically, the microbial diversity data and transcriptome data are available in the NCBI Sequence Read Archive under accession number PRJNA1296800.
