# Supplementary material for: Selenium nanoparticles and glutathione synergistically enhance salt tolerance in soybean via the jasmonic acid pathway and arbutin-regulated rhizosphere microbiota
Source: aBIOTECH. 2026 Jan 15;7(2):100022. doi: 10.1016/j.abiote.2026.100022 (PMC12973407; doi:10.1016/j.abiote.2026.100022)
Supplement: Multimedia component 2 [file mmc2.docx]

**Selenium nanoparticles and glutathione synergistically enhances salt tolerance in soybean via the jasmonic acid pathway and arbutin-regulated rhizosphere microbiota**

Yanxi Chen^a,b,1^, Shuai Zhao^b,1^, Xuanxuan Ma^a,1^, Li Ling^a^, Pengxin Du^a^, Xu Liu^a^, Yuhao Yao^a^, Qibin Ma^a^, Yanbo Cheng^a^, Yingxiang Wang^a^, Jian Wei^c,*^, Hai Nian^a,c,*^, Tengxiang Lian^a,*^

^a^ Guangdong Basic Research Center of Excellence for Precise Breeding of Future Crops, Guangdong Laboratory for Lingnan Modern Agriculture, Guangdong Provincial Key Laboratory for the Development Biology and Environmental Adaptation of Agricultural Organisms, South China Institute for Soybean Innovation Research, College of Agriculture, South China Agricultural University, Guangzhou, 510642, China

^b^ State Key Laboratory of Desert and Oasis Ecology, Xinjiang Institute of Ecology and Geography, Chinese Academy of Sciences, Urumqi, 830011, China

^c^ Northern Key Laboratory of Saline-tolerant Soybean Breeding, Ministry of Agriculture and Rural Areas, Jilin Agricultural University ,Changchun, 130118, China

^1^ These authors contributed equally

*Corresponding author email: liantx@scau.edu.cn

**Fig. S1.** Toxicological assessment and determination of optimal concentrations of SeNPs@CS and GSH for soybean growth. **A** Images showing soybean seed germination under various SeNPs@CS concentrations. **B** Germination rate of soybean seeds treated with different concentrations of SeNPs@CS. **C, D** Effects of different concentrations of SeNPs@CS (0, 5, 10, 20, and 40 mg/L) on aboveground (C) and belowground (D) biomass of soybean. **E, F** Effects of different concentrations of glutathione (0, 10, 25, 100, and 200 mg/L) on aboveground (E) and belowground (F) biomass. Different letters above bars indicate statistical differences among treatments (*P* < 0.05, LSD test).

**Fig. S2.** Phenotypic observation of soybean plants after foliar application of Se, GSH, and their combination. Representative side and overhead images of soybean plants after the third foliar spray (A, C). Representative side and overhead images of soybean plants after the fourth foliar spray (B, D).

**Fig. S3.** Changes in antioxidant components in soybean leaves under non-stress conditions. **A–J** SOD (A), POD (B), CAT (C), MDA (D), total antioxidant capacity (E), PAL (F), APX (G), MDHAR (H), GSH-Px (I), and GSH (J) levels in soybean plants after CK, Se, GSH, and SeG treatments. Different letters above bars indicate statistical differences among treatments (*P* < 0.05, LSD test).

**Fig. S4.** Effects of different treatments on root phenotypes, root length and belowground biomass of soybean seedlings under salt stress. **A** Root phenotypes of soybean seedlings subjected to different treatments under salt stress, including CK, SCK, Se, GSH, and SeG. **B** Root length of soybean seedlings under each treatment. **C** Belowground biomass of soybean seedlings under each treatment. Different letters above bars indicate statistical differences among treatments (*P* < 0.05, LSD test).

**Fig. S5.** Volcano plots of differentially expressed genes (DEGs) and metabolites (DEMs) in soybean. **A, B** Volcano plots showing DEGs in SCK vs. CK (A) and SeG vs. CK (B) (|Log₂FC| > 2, *P* < 0.05). **C, D** Volcano plots showing DEMs in SCK vs. CK (C) and SeG vs. CK (D) (|Log₂FC| > 1, *P* < 0.05).

**Fig. S6.** KEGG pathway annotation and enrichment analysis of transcriptomic in soybean. **A, B** KEGG enrichment analysis of upregulated differentially expressed genes (DEGs) in SCK vs. CK (A) and SeG vs. CK (B) comparisons. Colors indicate KEGG functional categories

**Fig. S7.** KEGG pathway annotation and enrichment analysis of upregulated differential metabolites under different treatments. **A-C** KEGG pathway annotation of upregulated differentially expressed metabolites (DEMs) in SCK vs. CK (A), SeG vs. CK (B), and SeG vs. SCK (C) comparisons. **D, E** Top 20 enriched KEGG pathways of upregulated DEMs in SCK vs. CK (F) and SeG vs. CK (G) comparisons. Dot size represents the number of metabolites and color indicates statistical significance.

**Fig. S8.** Volcano plots of differentially abundant ASVs in bacterial (A) and fungal (B) communities. Numbers indicate significantly upregulated (red) and downregulated (green) ASVs in each comparison.

**Fig. S9.** Correlation analysis between four microbial genera and the top 20 most strongly upregulated metabolites under SeG treatment (all with *P* < 0.05). Colors indicate the Pearson correlation coefficients, with red representing positive correlations and blue representing negative correlations. Metabolites and microbial genera are clustered based on similarity in correlation patterns.

**Fig. S10.** Relative abundances of three key metabolites in SCK and SeG groups. **A** 3-Methylglutaric acid (MGA). **B** Arbutin (Arb). **C** Baccatin III (Bac). Relative abundances were normalized to the mean value of the CK group for each metabolite. Statistical significance was assessed using a two-tailed Student’s t-test (* represents *P* < 0.05; ** represents *P* < 0.01; *** represents *P* < 0.001).

**Fig. S11.** Evaluation of individual and synthetic microbial treatments on soybean under salt stress. **A** Soybean plants subjected to salt stress with different treatments, including single microbial strains (RSB1, RSB2, RSS, RSP, and RSA), a synthetic bacterial community (SynB: RSB1, RSB2, RSS), a synthetic fungal community (SynF: RSP, RSA), and a combined bacterial–fungal community (Syn: RSB1, RSB2, RSS, RSP, RSA). The purpose of this experiment was to compare the effects of individual strains and synthetic microbial consortia on alleviating salt stress in soybean. **B-E** Quantitative results of the phenotypic images shown in Figure A, including plant height (B), root length (C), aboveground biomass (D), and belowground biomass (E) of soybean under different microbial treatments. **F** Soybean plants subjected to different synthetic microbial combinations to identify the optimal community. SynB-1 consists of RSB1 and RSS, while Syn-1 includes RSB1, RSS, RSP, and RSA. **G,H** Quantitative results from the phenotypic images in Figure F, including plant height (G) and aboveground biomass (H) of soybean under different synthetic microbial treatments. Different letters above bars indicate statistical differences among treatments (*P* < 0.05, LSD test).

**Fig. S12.** Colony morphology and phylogenetic analysis of individual members of the synthetic microbial community (Syncom). **A** Colony morphology of strain RSB1 on LB medium and its corresponding phylogenetic tree. **B** Colony morphology of strain RSS on LB medium and its corresponding phylogenetic tree. **C** Colony morphology of strain RSP on PDA medium and its corresponding phylogenetic tree. **D** Colony morphology of strain RSA on PDA medium and its corresponding phylogenetic tree.

**Table S1.** Top 20 significantly enriched pathways from the transcriptome-based GO enrichment analysis.

| GO: ID | Term Name | *P* Value | Gene Radio |
| --- | --- | --- | --- |
| GO:0020037 | heme binding | < 0.001 | 0.572 |
| GO:0046906 | tetrapyrrole binding | < 0.001 | 0.572 |
| GO:0016020 | membrane | < 0.001 | 0.386 |
| GO:0016491 | oxidoreductase activity | < 0.001 | 0.461 |
| GO:0055114 | oxidation-reduction process | < 0.001 | 0.465 |
| GO:0004497 | monooxygenase activity | < 0.001 | 0.579 |
| GO:0004713 | protein tyrosine kinase activity | < 0.001 | 0.462 |
| GO:0004672 | protein kinase activity | < 0.001 | 0.449 |
| GO:0006468 | protein phosphorylation | < 0.001 | 0.450 |
| GO:0004601 | peroxidase activity | < 0.001 | 0.603 |
| GO:0016684 | oxidoreductase activity, acting on peroxide as acceptor | < 0.001 | 0.600 |
| GO:0031224 | intrinsic component of membrane | < 0.001 | 0.402 |
| GO:0005506 | iron ion binding | < 0.001 | 0.511 |
| GO:0001071 | nucleic acid binding transcription factor activity | < 0.001 | 0.477 |
| GO:0003700 | transcription factor activity, sequence-specific DNA binding | < 0.001 | 0.477 |
| GO:0003824 | catalytic activity | < 0.001 | 0.397 |
| GO:0006979 | response to oxidative stress | < 0.001 | 0.592 |
| GO:0016209 | antioxidant activity | < 0.001 | 0.575 |
| GO:0016705 | oxidoreductase activity, acting on paired donors, with incorporation or reduction of molecular oxygen | < 0.001 | 0.515 |
| GO:0016021 | integral component of membrane | < 0.001 | 0.400 |

**Table S3.** Colony diameters of each member in Syncom under different treatments after 48 hours. Different letters indicate statistical differences among treatments (*P* < 0.05, LSD test).

| Group | RSP | RSS | RSB1 | RSA |
| --- | --- | --- | --- | --- |
| CK | 0.883cd | 0.492d | 1.233b | 3.967b |
| MGA-5 | 0.942bcd | 0.575bcd | 1.375ab | 4.217b |
| MGA-10 | 1.042abcd | 0.592bcd | 1.442ab | 4.008b |
| MGA-20 | 0.850d | 0.683ab | 1.600a | 4.083b |
| MGA-40 | 0.900cd | 0.592bcd | 1.325b | 4.283ab |
| Arb-5 | 1.125abc | 0.675abc | 1.225b | 3.983b |
| Arb-10 | 1.133abc | 0.692ab | 1.300b | 4.200b |
| Arb-20 | 1.108abc | 0.800a | 1.475ab | 4.233b |
| Arb-40 | 1.258a | 0.717ab | 1.483ab | 4.650a |
| Bac-5 | 1.183ab | 0.65abcd | 1.375ab | 4.283ab |
| Bac-10 | 0.967bcd | 0.5cd | 1.217b | 4.267b |
| Bac-20 | 1.00bcd | 0.558bcd | 1.433ab | 4.242b |
| Bac-40 | 0.950bcd | 0.6bcd | 1.408ab | 3.933b |

**Table S4.** Soil physicochemical properties in the field experiment.

| pH | Water-Soluble Nitrogen  (mg/kg) | Available Phosphorus  (mg/kg) | Effective Potassium  (mg/kg) | Organic Matter  (g/kg) | Total Salt Content  (g/kg) |
| --- | --- | --- | --- | --- | --- |
| 8.29 | 101.7 | 19.4 | 171.0 | 31.7 | 0.88 |
